# Urban Environmental Determinants and Spatiotemporal Patterns of Emergency Medical Service Response to Traumatic Injuries: A Five-Year Population-Based Study

**DOI:** 10.3390/ijerph23040434

**Published:** 2026-03-30

**Authors:** Akerke Chayakova, Oxana Tsigengagel

**Affiliations:** Department of Epidemiology and Biostatistics, Astana Medical University, Astana 010000, Kazakhstan; chayakova.a@amu.kz

**Keywords:** emergency medical services (EMS), geographic information systems (GIS), spatio-temporal analysis, urban health equity, sustainable urban development, wounds and injuries, prehospital care, Kazakhstan

## Abstract

**Highlights:**

**Public health relevance—How does this work relate to a public health issue?**

- Rapid urbanization in emerging economies creates structural barriers to equitable emergency medical service (EMS) access, directly impacting trauma survival rates.
- Traumatic injuries remain a leading cause of premature mortality, requiring optimized prehospital logistics to meet the “Golden Hour” clinical benchmark.

**Public health significance—Why is this work of significance to public health?**

- This study utilizes a robust five-year population-based dataset (*N* = 26,073) to identify a critical “Accessibility Paradox” where EMS delays are maximal during peak injury hours.
- The research provides the first comprehensive spatiotemporal audit of EMS performance in a major Central Asian capital, uncovering significant health equity gaps.

**Public health implications—What are the key implications or messages for practitioners, policy-makers and/or researchers in public health?**

- Health authorities should transition from population-based to proximity-based EMS deployment models, prioritizing satellite posts in underserved peripheral districts.
- Integrating real-time GIS analytics into urban health policy is essential for reducing preventable trauma-related disability and enhancing urban resilience.

**Abstract:**

Background: Timely prehospital management is critical for survival after traumatic injury. In rapidly growing metropolises, emergency medical service (EMS) systems often struggle to provide equitable care amid urban sprawl and traffic congestion. This study investigated spatiotemporal inequalities in trauma-related EMS response in a rapidly expanding capital city (Astana, Kazakhstan) to inform healthcare optimization and urban health equity. Methods: We analyzed a five-year population-based dataset of 26,073 trauma-related EMS calls recorded between 2020 and 2024. Spatial patterns were examined using Kernel Density Estimation (KDE) and Getis–Ord Gi* hotspot analysis. Road-network modeling assessed accessibility at 3, 5, and 10 min thresholds using a GIS-based network analyst framework. Results: Males accounted for 60.1% of utilization and had higher clinical severity (hospitalization rate: 45.5% vs. 40.3%, *p* < 0.001). Demand peaked at 20:00, coinciding with peak traffic. The mean total response time was 21.63 min, and only 16.9% of calls met the 10 min benchmark. Significant accessibility gaps were found in the Baikonur district (61.4% delay rate). Conclusions: The findings demonstrate that while the EMS system provides broad geographic coverage, it suffers from systemic spatiotemporal bottlenecks. Targeted infrastructure expansion in underserved peripheral districts and the implementation of dynamic deployment models are necessary to enhance urban health equity and reduce preventable mortality in expanding metropolitan areas.

## 1. Introduction

Trauma refers to sudden physical injury caused by an external force and remains one of the most pressing global public health challenges of the 21st century [1]. Beyond the immediate clinical impact, injuries impose a staggering socioeconomic burden, serving as a leading cause of premature mortality and long-term disability worldwide [2,3]. In economically developed nations, trauma is the second most prevalent cause of death, disproportionately claiming lives during an individual’s most productive working years [4,5]. Globally, approximately 4.4 million deaths, roughly 8% of total annual mortality, are attributed to injuries, with over 90% occurring in low- and middle-income countries where rapid access to specialized healthcare is often restricted [6,7].

The economic consequences of this crisis are equally profound. Road traffic crashes alone impose an annual global burden of approximately US$ 518 billion, consuming between 1% and 3% of the gross domestic product (GDP) in most nations [8]. This urgency is compounded by accelerating urbanization, economic instability, and increasing motorization. According to World Health Organization (WHO) projections, traumatic injuries will become the third leading contributor to the global burden of disease by 2030. Given the scale of casualties and associated costs, trauma has been aptly described as the “longest war of the modern world” [9,10]. Consequently, this research directly aligns with the United Nations Sustainable Development Goals (SDGs), specifically Goal 3.6, which aims to halve global traffic-related fatalities, and Goal 11.2, which emphasizes providing safe, resilient, and accessible transport systems for all.

Behind these staggering global statistics are thousands of individual lives interrupted during their most productive years [5]. In Kazakhstan, traumatic injuries occupy the fifth position in the structure of overall morbidity and mortality, yet they rank third in primary disability, highlighting a profound threat to the nation’s human capital [9]. In the management of acute trauma, the temporal element is the most critical determinant of survival and functional recovery [11,12]. The “Golden Hour” that thin line between life and death represents more than a medical benchmark; it is a universal promise of timely care that every citizen expects. The concept of the ‘Golden Hour’ underscores the importance of minimizing prehospital delay, although its empirical basis remains debated [13,14]. Consequently, ensuring the equitable geographic distribution and operational resilience of EMS is a fundamental requirement for modern urban sustainability [15,16].

International evidence suggests that prehospital time is not determined solely by distance, but by the interaction of settlement structure, traffic exposure, and the spatial distribution of EMS resources. A recent systematic review of 37 studies reported that most investigations found longer EMS response, transport, and total prehospital intervals in rural settings, while urban settings consistently demonstrated shorter prehospital times [17]. Likewise, a national Swedish trauma cohort showed that prehospital intervals increased with declining population density [18], while a large population-based study from the United States demonstrated that longer EMS response times were associated with higher rates of motor vehicle crash mortality [19]. Together, these findings indicate that delay is not merely an operational inconvenience, but a measurable determinant of trauma system performance.

Comparable evidence from other metropolitan healthcare systems further demonstrates that nominal citywide coverage may conceal substantial local inequities in effective access. In Beijing, greater ambulance and physician density were associated with shorter EMS response intervals and narrower urban–suburb disparities for out-of-hospital cardiac arrest [20]. In a separate isochrone-based accessibility analysis from the same city, mean population coverage within 10 min and 8 min decreased by 44.9% and 50.0%, respectively, during peak compared with midnight periods, underscoring the marked sensitivity of apparent service coverage to congestion [21].

In 2024, the Ministry of Health of the Republic of Kazakhstan reported that EMS providers attended to over 240,000 patients with traumatic injuries, a significant portion of which were casualties of road traffic accidents [22]. While national efforts have successfully reduced average urban response times to 15–20 min [23], citywide averages often mask deep-seated spatiotemporal inequalities. In rapidly expanding metropolises like Astana, unprecedented metropolitan expansion has created a “geography of risk.” Urban sprawl and traffic congestion create localized “pockets” of delayed care, where a person’s chance of survival may depend more on their neighborhood’s infrastructure than on the severity of their injury.

To address these complexities, injury epidemiology and EMS research have increasingly adopted geospatial methods to quantify Urban Health Equity. Prior studies have utilized spatial scan statistics (SaTScan) to detect statistically significant clusters of traumatic events [24], while others have employed Kernel Density Estimation (KDE) to produce continuous intensity surfaces that reveal how demand fluctuates by time of day [25]. Furthermore, hot spot statistics (e.g., Getis–Ord Gi*) allow researchers to distinguish persistent high-risk zones from random fluctuations, providing actionable data for infrastructure optimization [26].

Despite the global maturity of GIS-based injury surveillance, evidence from Central Asia and Kazakhstan in particular remains sparse. Most existing studies lack an integrated framework that combines multi-year spatiotemporal patterns with network-based accessibility modeling aligned with national performance benchmarks [27]. There is a critical need to understand how Astana’s unique urban morphology affects the “equity of access” to life-saving trauma care during its phase of rapid expansion. Astana serves as a unique laboratory for studying the ‘accessibility-urbanization mismatch’ common in rapidly expanding administrative centers across the Global South and Central Asia.

To address these gaps, this study aims to quantify spatiotemporal inequalities in trauma-related EMS demand and prehospital accessibility in Astana (2020–2024). By integrating road-network service-area modeling and statistically validated hotspot detection, we seek to identify high-burden underserved zones, providing a data-driven foundation for the strategic optimization of EMS infrastructure and the advancement of health equity in the capital.

## 2. Materials and Methods

### 2.1. Study Design and Data Sources

**Figure 1:**
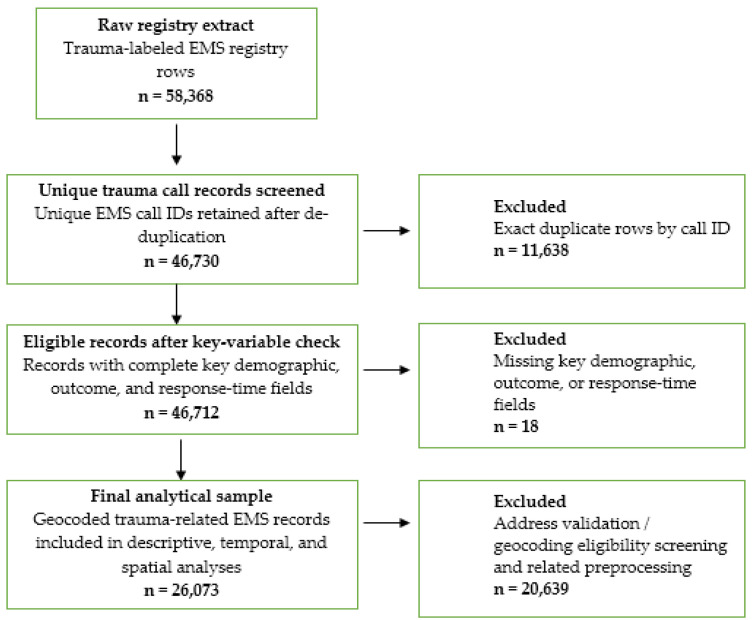
Data processing and cleaning flowchart.

We conducted a retrospective, population-based study of emergency medical service (EMS) utilization for traumatic injuries in Astana, Kazakhstan. The study period spanned 54 months, from 1 January 2020 to 30 June 2024. The primary dataset was obtained from the centralized digital archive of the Astana City Ambulance Station. The final analytical cohort consisted of anonymized records for *N* = 26,073 trauma-related calls (Figure 1). Each record included patient demographics (age and sex), temporal markers (exact date and time of the call, dispatch, arrival at the scene, and arrival at the receiving hospital), final EMS disposition, and address information for geocoding. Final disposition was categorized as hospitalized or Refusal of Hospitalization. For the purposes of this study, Refusal of Hospitalization was operationally defined as a trauma-related EMS encounter in which the patient was assessed on scene but was not transported for inpatient hospital care, as recorded in the final disposition field of the EMS registry.

The registry did not contain standardized trauma severity scores, such as the Injury Severity Score (ISS), Revised Trauma Score (RTS), or Glasgow Coma Scale (GCS), for the full hospitalized cohort. Therefore, formal severity stratification of admitted patients was not possible. The only available prehospital proxy of clinical status was the EMS-recorded field describing the patient’s condition at first contact.

### 2.2. EMS Activation and Dispatch Context in Kazakhstan

The call-to-dispatch interval in this study should be interpreted in the context of EMS organization in Kazakhstan. Emergency ambulance care is activated through 103 and can also be accessed through the unified emergency number 112, with medically relevant calls routed to ambulance dispatch. Kazakhstan operates a centralized EMS system in which dispatchers at ambulance stations receive the call, verify the location, assign an urgency category, provide telephone instructions when necessary, and allocate the responding team.

Under national rules, this dispatcher-processing stage has a target duration of 5 min [28]. Accordingly, the call-to-dispatch interval reflects the full call-processing period rather than a simple call-answer metric. Because the registry did not contain dispatcher-level reason codes, the specific causes of dispatch delay could not be identified directly. However, plausible contributors include dispatcher workload, mandatory triage and routing procedures, pre-arrival telephone instructions, and address clarification in a rapidly expanding urban environment.

### 2.3. Data Quality Assessment and Missing Data

A data-quality audit was performed before analysis. Blank fields were recoded as missing values. Exact repeated call identifiers were screened and removed during preprocessing. After de-duplication, only 18 records (0.04%) had missing information in at least one key variable used for the primary descriptive, temporal, or spatial analyses.

Missingness in the core analytic variables was negligible. Missing values for sex, disposition, dispatch time, scene-arrival time, and call-to-arrival time ranged from 0.002% to 0.03%, whereas age, urgency category, substation, and geocoded address variables were complete. Hospital-arrival time was missing more frequently (25.8%); however, this was primarily structural because many EMS encounters did not result in hospital conveyance. Therefore, records without hospital-arrival time were excluded only from analyses involving call-to-hospitalization intervals. Given the very low level of missingness in the primary study variables, no imputation was performed.

### 2.4. Statistical Analysis

Descriptive statistics were used to summarize demographic characteristics, EMS disposition, and service intervals. Continuous variables were presented as means with standard deviations and medians with interquartile ranges, as appropriate, while categorical variables were summarized as frequencies and percentages. Group comparisons were performed using standard inferential tests according to variable type and distribution.

In addition to descriptive and geospatial analyses, a multivariable logistic regression model was fitted to identify independent determinants of prolonged EMS response time. The dependent variable was defined as a call-to-arrival interval exceeding 10 min, in accordance with the national operational benchmark. Adjusted estimates were calculated for the selected demographic, temporal, and operational covariates included in the model.

### 2.5. Demographic and Temporal Analysis

To construct a comprehensive demographic profile, we analyzed the distribution of calls and subsequent hospitalizations across age cohorts and gender. Temporal patterns were evaluated at two scales: (i) Seasonal trends, analyzing fluctuations by month and season; and (ii) Diurnal cycles, examining hourly demand. EMS performance metrics (service intervals) were calculated as: Call-to-Dispatch (processing time), Dispatch-to-Scene (travel time), Call-to-Arrival (total response), and Call-to-Hospitalization (total prehospital time).

### 2.6. Geospatial Data Processing and Geocoding

Call addresses were converted into geographic coordinates using a multi-stage geocoding procedure. Records with ambiguous addresses were excluded. After processing, 26,073 geocoded points were integrated into a GIS environment (Figure 1). A topological road network model of Astana was constructed using OpenStreetMap (OSM) datasets, incorporating road hierarchies, surface types, and connectivity rules to ensure realistic routing.

### 2.7. Spatial Analysis and Modeling

Analysis was performed in ArcGIS Pro 3.1.0 using three methodologies, Kernel Density Estimation (KDE), to visualize the spatial intensity of trauma demand per square kilometer. To identify statistically significant spatial clusters. Confidence intervals (90%, 95%, and 99%) were used to validate the stability of high-demand (hot spot) and low-demand (cold spot) zones. Accessibility zones were modeled using the Network Analyst module for EMS stations and emergency hospitals. Modeling assumed a baseline speed of 50 km/h, with downward coefficients applied to secondary and residential roads. Service areas were defined at 3, 5, and 10 min intervals. Calls falling outside the 10 min zone were identified as “underserved pockets” at high risk for delayed care. To account for temporal variability, impedance factors for the road network were adjusted based on historical traffic flow patterns during morning and evening rush hours, ensuring the model reflects the ‘Accessibility Paradox’ identified in the results.

## 3. Results

### 3.1. Demographic Profile and Clinical Outcomes

An epidemiological analysis of *N* = 26,073 trauma-related EMS calls was conducted. The demographic assessment revealed a significant male predominance (60.1%, *n* = 15,661). Clinical outcomes were stratified by gender and treatment result, as shown in Table 1.

**Figure 2:**
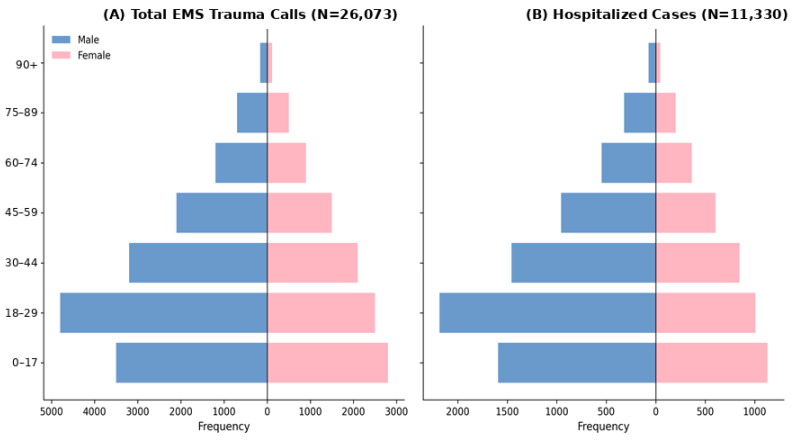
Population pyramids of trauma-related EMS utilization: (**A**) total calls; (**B**) hospitalized patients.

The population pyramids (Figure 2A) indicate that trauma demand is concentrated in the pediatric (under 18 years) and young adult (18–44 years) cohorts. A critical observation is the high rate of hospitalization refusal; 56.5% (*n* = 14,743) of trauma encounters were managed on-site without subsequent transport. Pearson’s chi-square test confirmed a statistically significant gender disparity in outcomes (*p* < 0.001): males were significantly more likely to be hospitalized than females (45.5% vs. 40.3%). As shown in Figure 2B, the peak demand for inpatient care is concentrated in the 10–30-year age group, likely reflecting exposure to high-energy unintentional injuries [25].

### 3.2. Temporal Trends and Performance Audit

**Figure 3:**
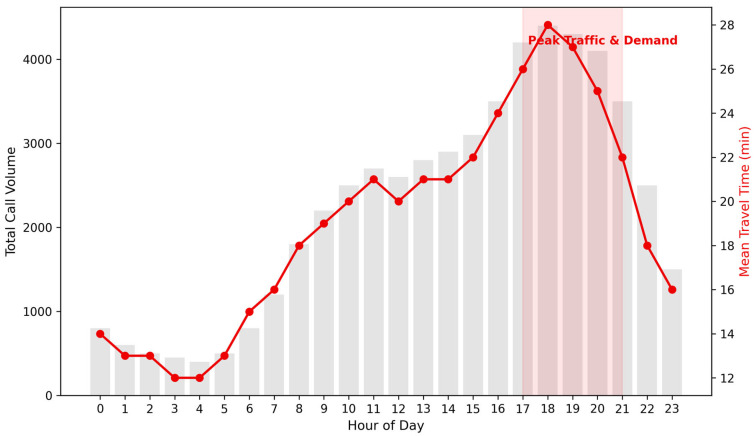
Diurnal rhythm of trauma demand vs. mean travel time.

Trauma demand exhibited pronounced seasonality, peaking during Spring (31.1%) and reaching its minimum in Winter (19.8%). Analysis of the diurnal rhythm (Figure 3) revealed a cyclical demand curve with activity reaching its absolute peak at 20:00 (approx. 4500 cumulative calls).

The EMS Performance Audit (Table 2) identified systemic bottlenecks in travel efficiency. While dispatch processing (Call-to-Dispatch) was relatively efficient (78.1% compliance with the <5 min benchmark), the mean travel time (Dispatch-to-Scene) was 15.02 min. Crucially, travel delays reached their maximum during the 17:00–21:00 interval, coinciding with peak urban traffic congestion. Overall, only 16.9% of trauma calls met the national 10 min total response benchmark.

### 3.3. Spatiotemporal Analysis and Accessibility Gap

**Figure 4:**
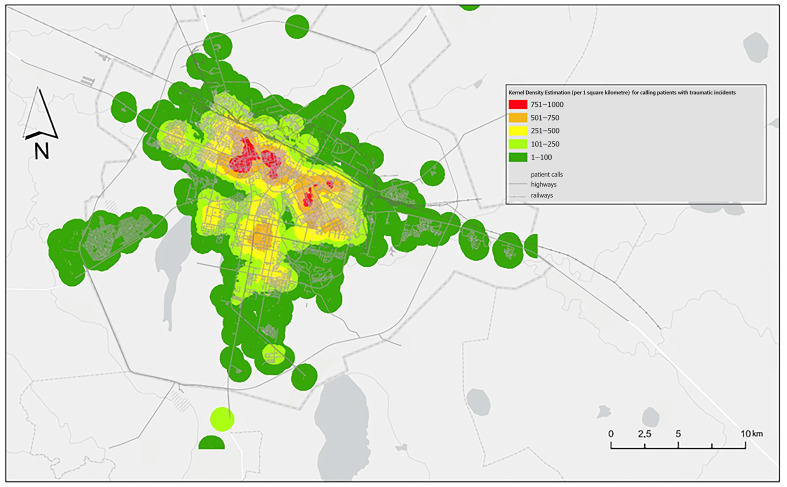
Spatial intensity of trauma incidents (KDE Analysis).

**Figure 5:**
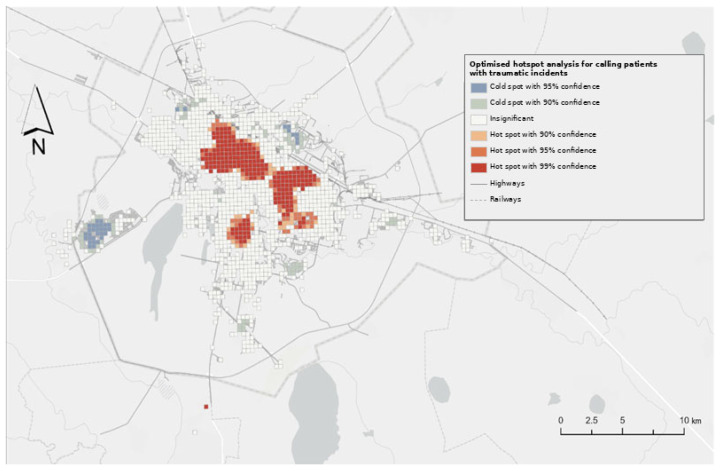
Optimized Hotspot Analysis (Getis-Ord Gi), identifying clusters of high demand (99% CI).

Kernel Density Estimation (KDE) (Figure 4) confirmed that trauma-related demand is heavily concentrated in the central urban core and along major arterial corridors. Hotspot Analysis (Figure 5) identified statistically significant clusters (99% CI) in the southern and central districts (Baikonur, Esil, and Saryarka), while the Nura district formed a persistent “cold spot.”

**Figure 6:**
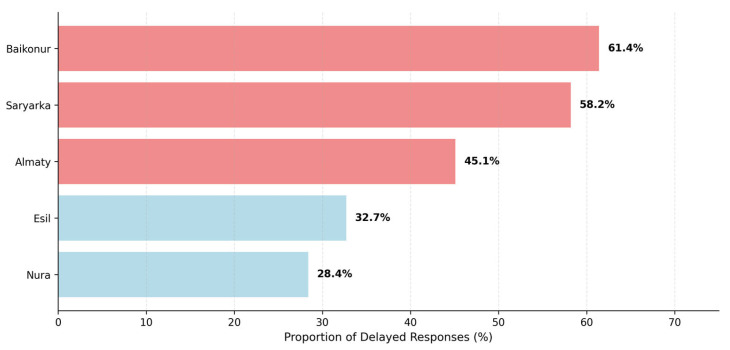
Delay Rate by District (>10 min travel time). The chart highlights the critical accessibility gap in the Baikonur and Saryarka districts. Note: Districts are color-coded to distinguish between the older “Old City” (red) and the newer administrative districts (blue).

The road-network modeling revealed a stark “equity gap” (Table 3). Although the majority of the city districts are within 10 min of an EMS post, 1.5% of calls (*n* = 394) originated from locations exceeding this critical threshold. These underserved pockets are primarily located in the northern peripheral zones of the Baikonur district, where the EMS arrival delay rate reached a critical high of 61.4% (Figure 6).

Table 4 presents the multivariable logistic regression analysis of factors associated with prolonged EMS response time, defined as a call-to-arrival interval >10 min. Urgency category was the strongest independent predictor. Compared with category 1 calls, the odds of prolonged response were significantly higher for category 2 (aOR 7.77, 95% CI 6.84–8.82), category 3 (aOR 40.53, 95% CI 35.97–45.68), and category 4 calls (aOR 125.67, 95% CI 71.03–222.35; all *p* < 0.001). Time of day also remained significantly associated with delay after adjustment. Relative to night-time calls, the odds of prolonged response were higher in the afternoon (aOR 1.21, 95% CI 1.10–1.33, *p* < 0.001) and evening (aOR 1.21, 95% CI 1.11–1.32, *p* < 0.001), whereas no significant difference was observed for morning calls. Female sex was associated with a modest but statistically significant increase in the likelihood of delayed response (aOR 1.12, 95% CI 1.05–1.19, *p* < 0.001). Age was also independently associated with the outcome, with higher odds observed among patients aged 0–17 years, 40–59 years, and ≥60 years compared with those aged 18–39 years. In addition, a clear temporal trend was identified across the study period. Compared with 2020, the adjusted odds of prolonged response increased significantly in each subsequent year and were highest in 2024 (aOR 2.40, 95% CI 2.11–2.73, *p* < 0.001). Overall, these findings indicate that delayed EMS response in trauma cases was shaped predominantly by operational and temporal characteristics of the service system.

## 4. Discussion

This study advances the evidence base on urban trauma-related EMS demand in Central Asia by providing a comprehensive spatiotemporal audit of Astana’s emergency infrastructure. By integrating demographic, temporal, and geospatial analyses, we demonstrate that trauma burden is not randomly distributed; rather, it is deeply embedded in the city’s urban morphology, mobility flows, and service accessibility [29,30]. Our findings reveal that behind the data points are thousands of individual lives interrupted during their most productive years, where the chance of survival is increasingly dictated by the “geography of risk” inherent in a rapidly expanding metropolis [31,32].

### 4.1. The Demographic Gap and Clinical Acuity

A primary finding of the demographic analysis is the concentration of trauma demand among the socially and economically active population, particularly men aged 10–44 years. This “severity gap” is underscored by the significantly higher hospitalization rate for males (45.5%) compared to females (40.3%, *p* < 0.001), suggesting that traumatic incidents involving men in Astana tend to be of higher clinical acuity. This pattern reflects higher occupational hazards in industrial and construction sectors and a greater prevalence of high-energy transport-related injuries among young adult males [6].

Notably, the high overall rate of hospitalization refusal (56.5%) warrants specific scrutiny. In this context, the ambulance service is effectively being utilized as a substitute for primary care, saturating dispatch capacity and delaying response to life-threatening cases [33,34]. To humanize this finding: every low-acuity call managed by an emergency crew represents a potential “invisible barrier” to a patient in critical need, highlighting the urgent necessity for integrated urgent care centers to protect the system’s life-saving mission.

### 4.2. The Diurnal Paradox and Safety Culture

The temporal analysis identified a pronounced “Accessibility Paradox”: EMS travel times reached their peak during the evening hours (17:00–21:00), precisely when service demand was at its highest. This pattern is most plausibly explained by urban traffic congestion, which substantially prolonged travel time compared with pre-dawn periods. In practical terms, this mismatch indicates that the system is most vulnerable at the very time when the need for rapid trauma response is greatest. A mean travel time of 28.0 min during the evening period therefore represents not only a temporal fluctuation, but a clinically relevant systems-level vulnerability.

Importantly, the prolonged interval from call to hospital admission is likely to reflect not only transport-related barriers, but also human and organizational factors across the prehospital–hospital interface. Prehospital trauma triage is an inherently complex decision-making process in which EMS personnel must balance speed against diagnostic certainty, stabilization needs, and destination choice [35]. After hospital arrival, additional delay may arise from ambulance offload delay related to emergency department crowding and access block [36,37]. Moreover, EMS-to-ED handover is vulnerable to several human-factor barriers, including conflicting priorities, lack of standardization, information loss, redundancy, technological limitations, and insufficient feedback between teams [38]. Patient- and family-related factors, including hesitation or refusal regarding transport, may also contribute in selected cases [39]. Although these mechanisms could not be measured directly in the registry, they provide a plausible explanation for why the mean call-to-admission interval remained prolonged despite the dominant role of spatial accessibility in the present analysis.

These findings are consistent with evidence from other countries and EMS models. In Beijing, isochrone-based accessibility analysis showed that peak-traffic conditions substantially reduced effective 10 min and 8 min coverage, while a mixed-method study from the same metropolitan system found that higher ambulance and physician density shortened response times and reduced urban–suburb disparities [21]. Likewise, in Isfahan, zone-based analysis of traffic-injury missions identified pronounced intra-urban variation in response intervals, particularly in dense and industrial areas [40]. Taken together, these studies suggest that the Accessibility Paradox observed in Astana is not an isolated local phenomenon, but part of a broader pattern seen in rapidly motorizing cities where nominal geographic coverage can be undermined by congestion and uneven resource distribution.

The multivariable model further clarifies this interpretation by showing that delay is not merely the product of random temporal fluctuation, but a structured expression of how urgency stratification, traffic-sensitive time windows, and operational geography interact within a rapidly expanding urban system. The exceptionally steep gradient across urgency categories suggests that lower-priority trauma calls are disproportionately exposed to system congestion and resource competition, a pattern consistent with recent evidence showing that EMS response performance is shaped by call priority, environmental conditions, and resource availability [41]. The persistence of afternoon and evening effects after adjustment reinforces our earlier conclusion that traffic is not simply a background inconvenience, but a central determinant of travel inefficiency in metropolitan trauma care; comparable studies have likewise identified roadway congestion and missions outside the routine operational catchment as major drivers of prehospital delay [42]. These findings indicate that avoidable delay is embedded in the organization of the urban EMS system itself and therefore requires dynamic redeployment during evening peak periods, closer surveillance of lower-priority calls, and periodic rebalancing of substation coverage to protect urban health equity.

From a health-systems perspective, addressing these bottlenecks is also a matter of quality management and institutional transparency. As previously emphasized in the Kazakhstani context, fostering a transparent safety culture, including the institutional willingness to identify and disclose medical errors, is essential for improving healthcare standards [43]. GIS-based auditing serves as a practical tool for identifying spatial inequalities and routing inefficiencies in EMS resource distribution [44,45]. Acknowledging that 74.9% of calls failed to meet the 10 min travel benchmark provides the empirical basis for accountability and supports the transition toward a more resilient, data-driven emergency care system.

### 4.3. Spatial Inequity and the “Old City” Barrier

Geospatial analysis yields the most actionable evidence for urban health policy, revealing that trauma hotspots (99% CI) are inextricably linked to high-density, transport-intensive environments [25]. However, the modeling of accessibility zones uncovered a stark “equity gap.” While citywide coverage appears effective on paper, 1.5% of incidents (*n* = 394) occurred outside the critical 10 min threshold.

These underserved “pockets” are primarily localized in the northern peripheral zones of the Baikonur district. This “Old City” area, characterized by narrower streets and aging infrastructure, suffers from the highest delay rates (61.4%). Comparable disparities have been documented in Busan, where hot spot areas had longer response times than cold spot areas and inequities increased toward the outskirts, and in Shanghai, where suburban districts had poorer EMS accessibility than central districts, particularly under peak traffic conditions. Similarly, in Beijing, urban–suburb disparities in EMS response time were linked to unequal distribution of ambulances and physicians [46,47]. From the perspective of Urban Health Equity, this spatial imbalance suggests that a resident’s postal code may determine their clinical outcome, a clear issue of social justice in urban planning. Achieving United Nations Sustainable Development Goal 11 (Sustainable Cities and Communities) requires that essential infrastructure remains resilient and accessible to all citizens, regardless of their neighborhood’s age or socioeconomic profile. Therefore, the transition from a population-based to a proximity-based EMS deployment model is not only a logistical necessity but a moral imperative for ensuring equitable health outcomes in a modern capital.

## 5. Limitations

This study has several limitations. First, the road-network modeling utilized a baseline speed of 50 km/h. While adjusted with coefficients for road hierarchy, it does not fully account for real-time traffic variability, extreme weather conditions (e.g., Astana’s severe winter blizzards), or the hospital “handover” time. Second, the dataset lacks clinical severity scores (e.g., ISS), making it difficult to definitively link response delays to mortality outcomes. Finally, as a single-city study, the findings may not be directly generalizable to rural Kazakhstan. However, the GIS-based methodology provides a replicable framework for national healthcare equity monitoring.

## 6. Conclusions

This study demonstrates that the path toward a Sustainable City must be paved with healthcare equity. While Astana’s trauma EMS system exhibits broad geographic coverage on a macro level, city-wide performance averages mask localized crises that disproportionately affect those in underserved peripheral districts. Our findings reveal that system efficiency is severely constrained by urban traffic congestion during evening peak hours, resulting in only 16.9% of calls meeting the critical 10 min response benchmark.

The integration of GIS technology is key to unmasking these spatiotemporal disparities. By identifying specific “accessibility gaps,” particularly in the northern Baikonur district, we provide more than just maps; we provide a blueprint for a more compassionate and resilient urban health system. Ensuring that the “Golden Hour” is achievable for every citizen—regardless of their neighborhood or the time of day is the ultimate benchmark of a sustainable and equitable capital. Our research serves as a diagnostic call to action for urban planners and health authorities to transition toward proximity-based, dynamic resource allocation to protect the lives of a rapidly growing metropolitan population.

## Figures and Tables

**Table 1:** Demographic Profile and Clinical Outcomes (*N* = 26,073).

Clinical Outcome	Males, *n* (%)	Females, *n* (%)	Total, *N* (%)
Refusal of Hospitalization	8529 (54.5%)	6214 (59.7%)	14,743 (56.5%)
Inpatient Admission	7132 (45.5%)	4198 (40.3%)	11,330 (43.5%)
Total Call Volume	15,661 (60.1%)	10,412 (39.9%)	26,073 (100%)
Statistical Significance			χ^2^ = 70.97, *p* < 0.001

Data are presented as absolute frequency (*n*) and row percentage (%). Percentages may not sum to 100% due to rounding.

**Table 2:** EMS Performance Audit: Prehospital time intervals and benchmark compliance.

Service Interval	Mean (SD), min	Median (IQR), min	Benchmark Compliance (%)
Call-to-Dispatch	6.29 (2.1)	5.1 (4.0–7.2)	78.1% within <5 min
Dispatch-to-Scene	15.02 (4.8)	13.8 (10.5–18.2)	25% within <10 min
Call-to-Arrival (Total)	21.63 (5.3)	19.5 (15.2–24.1)	16.9% within <10 min
Call-to-Admission	55.78 (12.4)	51.88 (41.7–64.2)	Target: “Golden Hour”

SD: standard deviation; IQR: interquartile range (25th–75th percentiles). The critical bottleneck is identified in the Dispatch-to-Scene interval, primarily driven by urban traffic congestion during peak demand hours.

**Table 3:** Spatial accessibility and regional inequity in EMS response by administrative district.

Administrative District	EMS Station Access (0–10 min)	Hospital Access (0–10 min)	Delay Rate (>10 min Travel)
Baikonur	98.2%	98.8%	61.4%
Saryarka	98.5%	99.5%	58.2%
Almaty	99.1%	99.8%	45.1%
Esil	99.6%	99.9%	32.7%
Nura	99.8%	99.9%	28.4%
Statistical Test			Kruskal-Wallis *p* < 0.001

Spatial accessibility was modeled using road-network service areas. The “Delay Rate” represents the proportion of actual calls where travel time exceeded the national 10 min threshold. Regional differences are statistically significant, highlighting a critical accessibility gap in the northern “Old City” districts.

**Table 4:** Independent determinants of prolonged EMS response time in multivariable logistic regression.

Variable	Category/Comparison	Adjusted OR (aOR)	95% CI	*p*-Value
Sex	Female vs. male	1.12	1.05–1.19	<0.001
Age group	0–17 vs. 18–39	1.14	1.07–1.23	<0.001
	40–59 vs. 18–39	1.10	1.01–1.19	0.026
	≥60 vs. 18–39	1.25	1.12–1.39	<0.001
Urgency category	Category 2 vs. 1	7.77	6.84–8.82	<0.001
	Category 3 vs. 1	40.53	35.97–45.68	<0.001
	Category 4 vs. 1	125.67	71.03–222.35	<0.001
Year of call	2021 vs. 2020	1.11	1.03–1.20	0.005
	2022 vs. 2020	1.70	1.55–1.86	<0.001
	2023 vs. 2020	1.53	1.41–1.66	<0.001
	2024 vs. 2020	2.40	2.11–2.73	<0.001
Season	Spring vs. winter	0.89	0.82–0.97	0.006
	Summer vs. winter	-	-	0.054
	Autumn vs. winter	0.86	0.79–0.95	0.002
Time of day	Morning vs. night	-	-	0.350
	Afternoon vs. night	1.21	1.10–1.33	<0.001
	Evening vs. night	1.21	1.11–1.32	<0.001
Day type	Weekend vs. weekday	0.92	0.87–0.98	0.011

The dependent variable was prolonged EMS response time, defined as call-to-arrival >10 min. Reference categories were male sex, age 18–39 years, urgency category 1, year 2020, winter, night-time, weekday.

## Data Availability

The data assessed and reported herein can be obtained from the authors upon reasonable request and following ethical and privacy principles.

## Abbreviations

The following abbreviations are used in this manuscript:

AMU	Astana Medical University
CI	Confidence Interval
df	Degrees of Freedom
EMS	Emergency Medical Services
GBD	Global Burden of Disease
GDP	Gross Domestic Product
GIS	Geographic Information System
HCF	Healthcare Facility
ICD-10	International Classification of Diseases, 10th Revision
IQR	Interquartile Range
ISS	Injury Severity Score
KDE	Kernel Density Estimation
MSHE RK	Ministry of Science and Higher Education of the Republic of Kazakhstan
NpJSC	Non-profit Joint-Stock Company
OSM	OpenStreetMap
SD	Standard Deviation
SDG	Sustainable Development Goals
SRN	Street and Road Network
WHO	World Health Organization
